# Malaria and COVID-19: unmasking their ties

**DOI:** 10.1186/s12936-020-03541-w

**Published:** 2020-12-23

**Authors:** Mogahed Ismail Hassan Hussein, Ahmed Abdalazim Dafallah Albashir, Omer Ali Mohamed Ahmed Elawad, Anmar Homeida

**Affiliations:** Faculty of Medicine, University of Gezira, Wad Medani, Sudan

**Keywords:** Malaria, COVID-19, ACE2, Hydroxychloroquine and chloroquine, Malaria and COVID-19 syndemics, Malaria service

## Abstract

The incidence and mortality of COVID-19, according to the World Health Organization reports, shows a noticeable difference between North America, Western Europe, and South Asia on one hand and most African countries on the other hand, especially the malaria-endemic countries. Although this observation could be attributed to limited testing capacity, mitigation tools adopted and cultural habits, many theories have been postulated to explain this difference in prevalence and mortality. Because death tends to occur more in elders, both the role of demography, and how the age structure of a population may contribute to the difference in mortality rate between countries were discussed. The variable distribution of the ACEI/D and the ACE2 (C1173T substitution) polymorphisms has been postulated to explain this variable prevalence. Up-to-date data regarding the role of hydroxychloroquine (HCQ) and chloroquine (CQ) in COVID-19 have been summarized. The article also sheds lights on how the similarity of malaria and COVID-19 symptoms can lead to misdiagnosis of one disease for the other or overlooking the possibility of co-infection. As the COVID-19 pandemic threatens the delivery of malaria services, such as the distribution of insecticide-treated nets (ITNs), indoor residual spraying, as well as malaria chemoprevention there is an urgent need for rapid and effective responses to avoid malaria outbreaks.

## Background

Malaria is a parasitic infection, caused by parasites of the genus *Plasmodium* and transmitted by *Anopheles* mosquitoes, that leads to an acute life-threatening disease and poses a notable global health threat. According to the World Health Organization (WHO) data of 2018, about 228 million cases of malaria and 405,000 deaths were reported worldwide, with Africa displaying the greatest number of cases and the highest mortality [1].

In December 2019, a new coronavirus was identified to be responsible for many pneumonia cases in Wuhan, a city in the Hubei Province of China. The number of cases has then increased exceedingly in China, and then all over the world causing an epidemic. The virus is now named severe acute respiratory syndrome coronavirus 2 (SARS-CoV-2). The disease was initially reported to the WHO in December 31, 2019, and the COVID-19 epidemic was declared a global health emergency in January 30, 2020, then a global pandemic in March 11, 2020 [2, 3].

While malaria and COVID-19 can have similar presentation, common symptoms they share include but not limited to: fever, breathing difficulties, tiredness and acute onset headache, which may lead to misdiagnosis of malaria for COVID-19 and vice versa, particularly when clinician relies mainly on symptoms.

COVID-19 disturbs the continuity of the Population Services International and the Global Malaria Community, such as seasonal malaria chemoprevention (SMC) and insecticide-treated bed nets (ITNs) distribution. Malaria testing and treatment are also disturbed due to the risks faced by health workers who provide health care services during the pandemic. Decision-makers will need to make difficult choices to ensure that COVID-19 and other urgent, ongoing public health problems- including malaria endemics—are addressed while minimizing risks to health workers and communities. As established at the 2018 Astana Global Conference on Primary Health Care, the community level is an integral platform for primary health care, key to the delivery of services and essential public health functions, and to the engagement and empowerment of communities in relation to their health. The community-based activities towards supporting the continuity of essential services, such as malaria prevention, diagnosis and treatment, with its distinctive capacities for health care delivery and social engagement, have a critical role to play in the response to COVID-19 and are essential to meet people’s ongoing health necessities, particularly for the most vulnerable population. Prevailing delivery approaches will need to be adapted as the risk–benefit analysis for any given activity changes in the context of a pandemic.

## The low prevalence of COVID-19 in malaria-endemic countries

The spread of COVID-19 in Africa is considered less than expected. As of 5:33 pm CEST, 1 August 2020, a total of 17,396,943 COVID-19 confirmed cases and 675,060 deaths have been reported worldwide by the WHO [4]. Of notice, the confirmed number of patients in Africa was only 788,488. This represents about 4.53% of the confirmed cases globally. The number of confirmed cases is relatively low in countries such as Mozambique (1864 confirmed cases and 11 deaths), the Democratic Republic of the Congo (9069 confirmed cases and 214 deaths), Nigeria (43,151confirmed cases and 879 deaths), Uganda (1154 confirmed cases and 3 deaths), Côte d’ Ivoire (16,047 confirmed cases and 102 deaths), and Niger (1136 confirmed cases and 69 deaths), especially where malaria is common [3]. Nigeria (25%), the Democratic Republic of the Congo (12%), Uganda (5%), Côte d’ Ivoire (4%), Mozambique (4%) and Niger (4%) represent more than half of global number of malaria cases [1]. The total number of citizens in these six countries is approximately 400 million, with a cumulative number of confirmed COVID1-19 cases being 72,421. As a comparison, in the WHO European region (~ 741 million of people), the cumulative number of COVID-19 patients during the same period, is 3,357,465. Meanwhile, 4,456,389 cases of COVID-19 have been reported in the USA (~ 327 million people). According to the Africa Centers for Disease Control and Prevention, a total of 736,288 COVID-19 cases and 15,418 deaths (CFR: 2.1%) have been reported in 54 African countries. This is 5% of all cases reported globally.

The angiotensin-converting enzyme 2 (ACE2), hydroxychloroquine (HCQ) and chloroquine (CQ), interferons and the neutralizing antibodies have been postulated to play roles in the low prevalence of COVID-19 in malaria-endemic countries. The Africa Centers for Disease Control and Prevention (Africa CDC) has pioneered efforts to expand laboratory services in the Continent. This was done through the African Task Force for Coronavirus Preparedness and Response (AFACTOR)—a coalition between the African Union (AU), the AU member states, the WHO regional office for Africa, and other stakeholders. AFACTOR has been instrumental in this impressive achievement, promoting coordination and alignment for evidence-based public health action. Inspite of all this effort, there are various areas that underscore persisting weaknesses in laboratory systems and networks. While maintaining its robust mobilization against COVID-19, faced with a public-health crisis of unprecedented scale, Africa has demonstrated solidarity and unified leadership in responding quickly. Nevertheless, Africa is still on a trajectory to reach-out global regions more affected by COVID-19, especially at surveillance and the test-trace-isolate-treat strategy [5].

## The participation of age structure variations

The numbers of older populations and the pace of aging differ broadly between and within regions. Typically, the more developed regions have higher proportions of their populations in older age groups than do developing ones. Population age structure has its role in the remarkable variation in the COVID-19 vulnerability and fatalities across countries. The COVID-19 mortality risk is highly focused at older ages, especially those 80 + years of age. The high mortality of COVID-19 in Italy was unexpected, given the affected region’s health and wealth. Italy is one of the oldest populations, with 23.3% of its population over 65 years of age, compared to 12% in China [6]. The youngest population is found in Africa, with a median age of < 20 years when compared with Europe and the USA (median age > 38 years), may have contributed to the low numbers of severe COVID-19 cases and mortality rates [6, 7]. This is a plausible reasoning, although its role may be less because of other pervasive underlying factors, such as malnutrition, risky livelihood and cultural factors brought about by the characteristics of the informal economic sectors they work in, as well as overcrowding within urban settlements. A study evaluating the impact of population age on COVID-19 fatalities found a standardized mortality ratio, which uses age-specific case fatality rates CFRs, that was fourfold less in Africa when compared to Europe and North America and > twofold less when compared to Asia and South America [8]. The young COVID-19 patients are usually asymptomatic or have mild symptoms that can be missed by targeted surveillance and testing; that is why the contribution of this factor may be better assessed by administering well-designed prevalence studies to determine the extent of SARS-CoV-2 infections in various contexts (urban, peri-urban, and rural) within the continent.

## The role of ACE2 in malaria and COVID-19

**Fig. 1:**
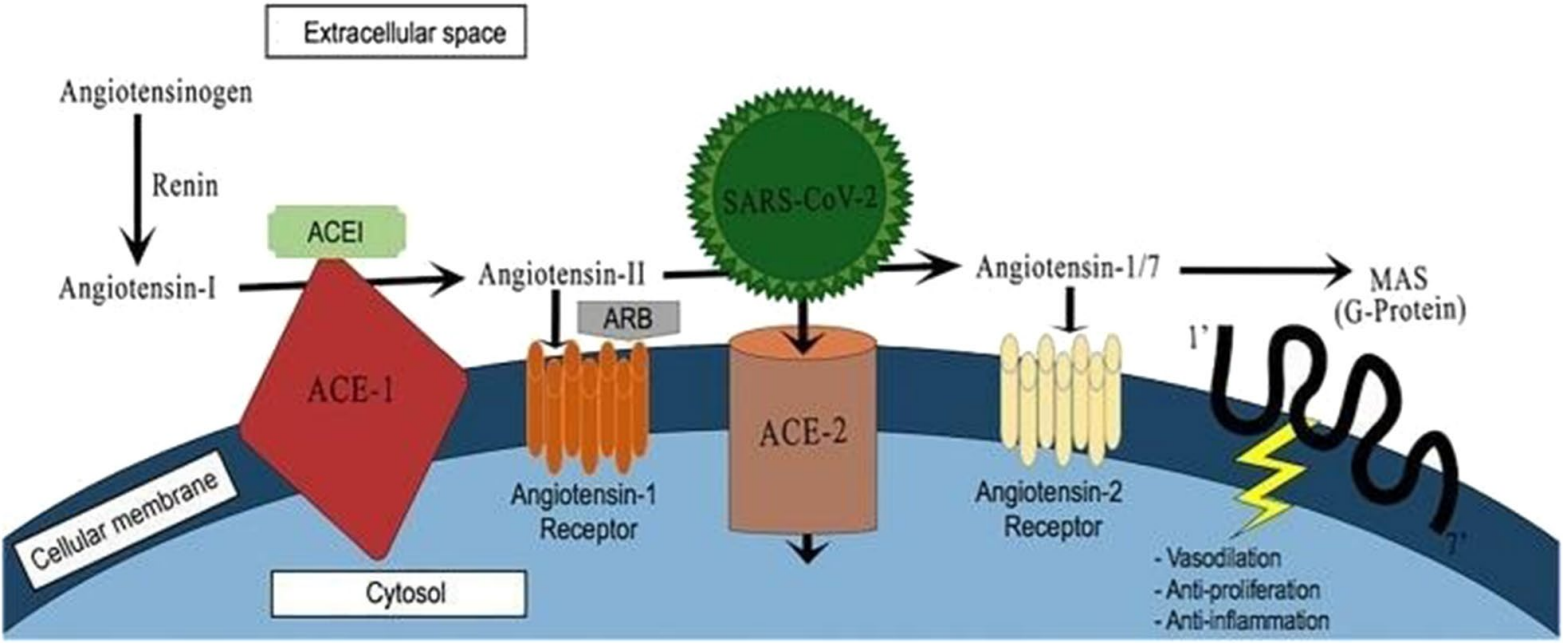
It shows the RAAS pathway, mechanism of action of ACEIs and ARBs, and the use of ACE2 receptors by SARS and SARS-COV2 for host cell entry. *ARBs* angiotensin receptor blockers, *ACEIs* angiotensin-converting enzyme inhibitors

Studies have revealed that SARS-CoV-2 uses the angiotensin-converting enzyme 2 (ACE2) receptor to enter the host cells (Fig. 1). ACE2 is a type I transmembrane amino-peptidase that is mainly anchored at the apical surface of cells of the gastrointestinal system, heart, kidneys, blood vessels and is highly expressed in the heart and type II alveolar cells of the lungs [9]. In addition to the membrane-bound form, there are soluble forms in the plasma and urine. It was first discovered in 2000 as an ACE homologue and shares approximately 42% homology with angiotensin-converting 1 (ACE1). It is capable of producing the lung-protective Ang-(1–7) from angiotensin II (ANG II) and converting angiotensin I to angiotensin(1–9) [10] (Fig. 1). If the ACE2 receptor activity underwent downregulation, ANG II-the substrate for ACE2- will then accumulate. Accumulated ANG II will then increase neutrophils aggregation and enhances vascular permeability. An exacerbation of pulmonary oedema and ARDS will eventually ensue [11].

Reports that ANG II impairs *Plasmodium* development were initially described in the sexual cycle of *Plasmodium gallinaceum*, an avian malaria parasite. ANG II decreases the buildup of sporozoites in mosquitoes’ salivary glands by directly disturbing the parasite membrane [12]. Other studies have illustrated a clear protective effect of Ang II in malaria. The ACE1 enzyme is distinguished by a genetic deletion/insertion polymorphism in intron 16. The presence of this (D/I) polymorphism is linked to changes in the concentration of both circulating and tissue-bound forms of ACE. When the presence of D allele is dominating, this is associated with reduced expression of ACE2 receptor. In a genetic association study, it has been shown that the presence of D-allele of ACEI/D polymorphism, which enhances Angiotensin II production, is associated with a mild pattern of malaria. The reduced expression of ACE2 receptor in populations with this polymorphism may play a protective role against COVID-19. Globally, the ACE I/D allele ratio is variable [13]. The ACEI/D polymorphism and the consequent increase in Ang II plasma levels were demonstrated in people with African genetic background. In a recently published paper, the log-transformed prevalence of COVID-19 infection was seen to be inversely related to the ACE D allele frequency: log (prevalence; number of cases/106 inhabitants) = 6.358–0.079 (D-allele frequency, %), r2 = 0.378; p = 0.001 [14]. About 38% of the variability of the prevalence can be justified by the relative frequency of the ACE1 D-allele. Likewise, a significant correlation could be noted between COVID-19 caused mortality (Spearman r =  − 0.510, p = 0.01). The two Asian countries which were initially severely hit by the virus, China and Korea, are marked by low D allele frequencies. As previously mentioned, the ACE2 receptor is used by SARS-CoV- 2 for host cell entry and the D/I polymorphism shows an important geographical variation [15]. Therefore, the variability in D/I genotype distribution could partly explain the variable prevalence of the COVID-19 infection between continents.

Another concerning polymorphism is the ACE2 polymorphism (C1173T substitution), which reduces ACE2 receptor expression in the presence of the T allele and consequently increases ANG II. This reduced expression results in an increase in Angiotensin II as ACE2 receptors are responsible for converting Angiotensin II to Ang-(1–7). Again, this phenomenon may potentially explain the various distribution of COVID-19 among countries. However, the available data is still scarce and additional genetic studies are needed to confirm this theory.

## The roles of interferons and the neutralizing antibodies in malaria and COVID-19

Reports from several studies have shown that there were interferons produced by lymphocytes as an immune response to infection by several strains of malaria, these interferons have both in vitro and in vivo efficacy against the coronaviruses responsible for SARS, MERS and COVID-19 [16–18]. Malaria patients develop antibodies against *Plasmodium* specific antigens. Some of these IgG antibodies target Glycosylphosphatidylinositol (GPI) molecules, which anchor some membrane proteins of *Plasmodium* species. Although the previous infection of malaria is not fully protective, as evidenced by repeated infections encountered by individuals in malaria-endemic regions, the severity of clinical presentation in such “semi-immune” subjects is less than in non-immune [19]. GPI acts mainly through stimulating leukocytes, triggering the release of pro-inflammatory cytokines and stimulating the expression of adhesion molecules via Toll-like receptors 2 and 4. Anti-GPI antibodies may neutralize these toxic effects of *Plasmodium* GPI. Also, SARS-CoV-2 has various glycoproteins (GPs): membrane GPs, spike GPs and GPs that have acetyl esterase and haemagglutination features. These GPs could be identified by the anti-GPI antibodies resulting in protection against virus infection or inducing a milder disease pattern [20].

## The use of hydroxychloroquine and chloroquine in COVID 19

Some scientists attribute the inverse relationship between COVID 19 and malaria to the wide use of hydroxychloroquine (HCQ), chloroquine (CQ) and other anti-malarial drugs in countries that are endemic for malaria [21]. It is important to point out that HCQ and CQ efficacy in the treatment of coronavirus diseases has been studied since the first SARS epidemic [22, 23]. Some old studies highlighted the importance of HCQ in the management of SARS-COV2 and suggested that 400 mg of HCQ per day for 10 days can be used as an optimal regimen [24]. However, recent clinical studies have considered HCQ for SARS-Cov-2 to be used at a dose of 400 mg PO twice in the first day as a loading dose, followed by 200 mg every 12 h for a period of 4 days [25]. On 22 May 2020, a reported clinical trial concluded that HCQ or CQ use in COVID 19 carries more risks than benefits [26]; however The Lancet Journal editor has indicated that the article will be withdrawn because of data concerns [27, 28]. It is important to note that the use of CQ and its derivatives is still a common practice in countries where malaria is endemic, despite drug resistance and WHO recommendations [21, 29]. All these factors are the reason why some scientists see the use of anti-malarial medication as unintentional chemoprophylaxis against SARS-CoV-2.

Some scientists argued that the use of CQ and its derivatives as a prophylactic medication could slow down coronavirus spread among healthcare workers. They considered that the wide availability of the medication makes it a feasible and practical option once its efficacy is scientifically proved [22, 29]. In vitro studies showed that CQ inhibits SARS-CoV replication in both infected and healthy cells, pointing to its prophylactic activity [23]. Keeping in mind that HCQ and CQ share a common molecular mechanism, it is very likely that they share a common effect on disease prevention and progression [30]. Meticulous and more extensive in vivo and in vitro studies are obviously needed. Clinical trials are already investigating HCQ use as a preventive measure in health care workers and family home isolated COVID patients [31, 32]. Recently published double-blind clinical trial (n = 821) found no difference between HCQ (n = 414) and placebo in terms of COVID prevention in the setting of immediate use in symptoms free individuals who have a high-risk exposure, note that the incidence of COVID-19 did not differ between groups [33]. The U.S. National Institute of Health is conducting phase 2b placebo-controlled trial to assess the effect of outpatient HCQ treatment on the rate of death and hospital admissions [34]. A randomized trial in the UK (UK RECOVERY) assigned 1542 patients to receive HCQ and 3132 to the standard care, the primary end point is 28-day mortality. Early data suggests no difference between HCQ and standard care in terms of the primary endpoint or any other outcome [35].

**Fig. 2:**
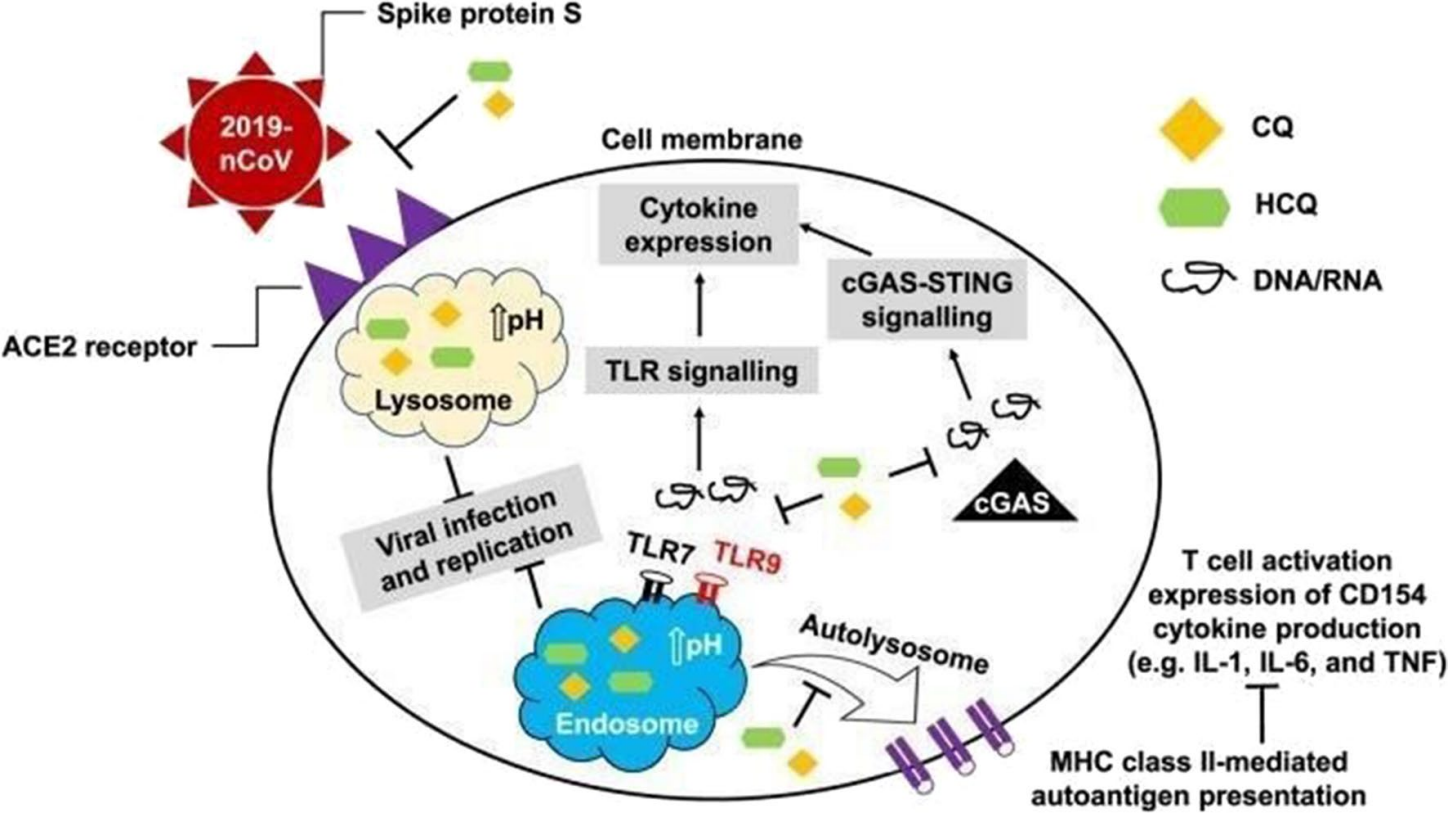
Hydroxychloroquine (HCQ) and chloroquine (CQ) interfere with T cell activation and prevent co-stimulatory signals and cytokines release. They inhibit lysosomes, prevent membrane fusion and antigen presentation rendering T cell inactivated. They also inhibit the virus binding to ACE2 receptors and prevent viral entry to the cell. *TLRs* toll-like receptors, *MHC* major histocompatibility complex, *cGAS* cyclic GMP-AMP synthase

The body fights viral infections by cellular immunity, antigen-presenting cells (APCs) process the foreign virus via major histocompatibility class II (MHC II). The antigen-presentation is a cytoplasmic process and is essential for T cell activation which also plays a major role in the disease pathophysiology [36]. The anti-malarial medications HCQ and CQ may interfere with this pathway and reduce T cell activation and prevent co-stimulatory signals and cytokines release [37]^.^ HCQ has high pH and when it enters the host cells it increases cellular pH. High pH inhibits lysosomes and by doing so, antigen presentation will not be possible and T cell will not be activated [30]. Moreover, altered cytoplasmic pH will interfere with toll-like receptors (TLR) particularly TLR7 and TLR9 [38, 39]. Toll-like receptors are linked to interferon genes stimulation via STING pathway, so HCQ interfere with this pathway and result in an attenuated inflammatory response, these mechanisms have led to the hypothesis that HCQ may be beneficial in the setting of cytokines release syndrome (CRS) which occur due to massive immune activation in the setting of SARS-Cov-2 infection [30]. Immune modulation is not the only way through which HCQ and CQ may act against coronavirus infection, they also inhibit the virus binding to its target host receptors (ACE2) as well as the membrane fusion (Fig. 2) [30]. They can also prevent receptor virus interaction by altering the glycosylation of ACE2 receptors thus reducing the binding affinity between the cell receptors and the virus spike proteins [30]. By doing so, HCQ and CQ prevent viral entry to the cells. One step after receptor binding, SARS-Cov-2 virus uses endosomes to enter host cells, these cellular structures are characterized by a low pH, HCQ and CQ become concentrated in endosomes once they enter the cells and the pH of the endosome will be increased, this will halt down the endosomes and viral fusion process [23]. The membrane fusion process between host cells and the virus is also affected by the increase in lysosomal pH mediated by CQ and HCQ. This fusion process is activated by lysosomal proteases which cleave the virus spike proteins [38]. Lysosomal proteases activity decreases in the setting of high pH [40, 41]. In the setting of inappropriately high pH, cell organelles will not be a proper place for viral replication [42].

In summary, the wide use of CQ and HCQ in malaria-endemic countries may be responsible for the inverse relation between COVID 19 and malaria prevalence. This is because these medications may have both preventive and curative effects against SARS-CoV-2 virus through three main mechanisms: (i) Halting the disease progression and inhibiting cytokines storm by reducing T cell activation; (ii) Changing cellular pH and preventing viral replication;(iii) CQ have demonstrated antiviral activity in both infected and healthy cells which may apply also to HCQ. Keeping in mind that HCQ is not intended to be used as anti-malarial in Africa, and CQ has been withdrawn from the list of anti-malarial in most African regions and was replaced by artemisinin-based combination therapy, which does not have any evidence of in vivo activity against COVID.

## The similarity between malaria and COVID-19 symptoms

The clinical features of COVID-19 ranged from asymptomatic to severe symptoms. The symptoms include fever, cough, sputum production and fatigue. They may also include headache, arthralgia, myalgia, nausea and vomiting [43]. Comparatively, malaria patients usually present with fever, headache, chills and sweating, other symptoms may include fatigue, arthralgia, myalgia, nausea, vomiting, and diarrhoea [44]. Due to the similarity of symptoms between malaria and COVID-19, especially fever, difficulty in breathing, fatigue and headache of acute onset, a malaria patient may be misdiagnosed as COVID-19 and vice versa. Moreover, complications like acute respiratory distress syndrome (ARDS), septic shock, and multi-organ failure can also occur in both malaria and COVID-19. The first step to identify a COVID-19 patient is the symptomatic screening, which consists of shortness of breath, fever, dry cough, sore throat, headache and myalgia in a high-risk patient like healthcare workers or patients with a history of contact with a confirmed COVID-19 case. These screening approaches can fail to catch about 50% of the COVID-19 patients even in countries with excellent health systems [45]. Unquestionably, the high index of suspicion is currently skewed towards COVID-19 regarding the alertness locally, regionally and internationally. Currently, people with fever may be tested for COVID-19 and then sent home due to a negative result, ignoring the possibility of malaria. Overlooking a malaria case can lead to fatal malaria complications. In contrast, febrile patients may get tested for malaria when they actually have COVID-19 infection. One single case of COVID-19 has the potential to affect up to 3.58 susceptible individuals. A third possible scenario is that a patient may have COVID-19 and malaria co-infection and the diagnosis and treatment of one of them may lead to missing the other.

## Malaria and COVID-19 syndemics

Syndemics occur when two or more coexisting infections have a harmful interaction, that is when coinfection occurs, it leads to a worse overall outcome than for either individual infection. For example, malaria and Epstein–Barr virus (EBV) coinfection can lead to Burkitt’s lymphoma as malaria contribute to B-cell proliferation and increasing EBV loads. Another example, HIV-infected people encounter a greater frequency of severe malaria and increased HIV viral load following infection with *Plasmodium falciparum*. Several parasites-HIV coinfections are associated with increased HIV viral load and worsened immunosuppression.

The onset of symptoms in COVID-19 is generally 4–5 days after infection, although it can be as late as 14 days [46]. Approximately, a week after the development of symptoms, some patients will develop an acute worsening, with a pronounced systemic increase of inflammatory mediators and cytokines, the cytokine storm [47]. This storm is characterized by considerably increased levels of interleukins (IL) and tumour necrosis factor (TNF) and is associated with the development of acute respiratory distress syndrome (ARDS) [48]. Among 72,314 cases reported from China, 14% were rated as severe and 5% were critical (respiratory failure, septic shock, and multiple organ dysfunction or failure) [49]. In malaria, merozoites of the Plasmodium enter erythrocytes and mature into schizonts. When schizonts break up, releasing new merozoites into the bloodstream, this causes fever and other manifestations of malaria. Hence, the clinical features of malaria are due to release of pro-inflammatory cytokines including TNF, interferon-gamma, IL-6, and IL-12 [50]. Studies in malaria-endemic countries have found that it is crucial to have a balance between a host pro-inflammatory, Th1 response (e.g., TNF, IL-6, IL-12, and interferon-gamma) and anti-inflammatory, Th2 response (IL-4, IL-10, and others) [51, 52]. The excessive pro-inflammatory responses are often the cause of severe manifestations of malaria. The same appears to be true in at least some cases of COVID-19 [53], proposing that a coinfection that also leads to excess proinflammatory responses might result in more severe manifestations and poor prognosis.

Respiratory distress, seen in up to 40% of children and 25% of adults with severe *P. falciparum* malaria, has many causes including severe anaemia, metabolic acidosis, cytoadherence of infected erythrocytes in the pulmonary vasculature, and co-infections with pneumonia-causing pathogens [53]. The clinical spectrum ranges from mild upper respiratory symptoms to fatal acute respiratory distress syndrome (ARDS). ARDS occurs in 5–25% of adults and 29% of pregnant women with severe *P. falciparum* malaria, but it is rarely seen in young children with malaria and patients with *P. vivax* malaria [54]. ARDS, both in COVID-19 and malaria, is due to inflammatory cytokine-mediated increased capillary permeability or endothelial damage, with the resultant of major alveolar damage [53]. Given this situation, SARS-CoV-2—*Plasmodium* spp. coinfections may lead to rapid deterioration and poor prognosis. Of note, the inflammatory-induced alveolar damage seen in malaria-induced ARDS continues even after treatment and parasite clearance [53]. Therefore, coinfection may result in severe COVID-19, and clinicians should keep this in mind.

Many viral infections, including SARS-CoV-2, produce a pro-coagulant state via inducing tissue factor expression, causing endothelial dysfunction and Toll-like receptor activation, and elevating von Willebrand factor levels [55, 56]. Elevated D-dimer and fibrin degradation product levels and prolongation of prothrombin time are associated with a poor prognosis [55]. The hypercoagulable state in COVID-19 is associated with a high rate of venous and arterial thrombotic complications (e.g. pulmonary embolism) [57, 58]. COVID-19 increases the risk for developing disseminated intravascular coagulation (DIC) [43, 59]. Autopsy findings have revealed both pulmonary haemorrhage and thrombosis [60]. Thrombocytopenia, which is also a potential feature of COVID-19, is deemed to occur due to excessive activation of the coagulation cascade, leading to platelet activation and consequent consumption [55]. It is associated with a poor prognosis in COVID-19 [61].

Comparatively, malaria is also associated with a pro-coagulant state. The TNF and IL-6 lead to activation of the coagulation cascade, which is proportionate to disease severity [62]. Malaria is usually associated with micro-thrombotic complications, however, thrombosis of larger vessels including cerebral venous thrombosis and pulmonary embolism have been reported [63, 64].

Thrombocytopenia occurs in 60–80% of malaria patients [60]. Bleeding and DIC occur only in severe malarial cases accompanied by coagulopathy, and they are associated with high mortality [62, 65]. The release of tissue factor from damaged vascular endothelial cells and the lysis of activated platelets produce a coagulant state, similar to the postulated mechanism in COVID-19 [65]. Accordingly, SARS-CoV-2-*Plasmodium* spp. coinfection may result in a more severe degree of coagulopathy and more severe disease than with either one alone.

This, therefore, necessitates enhancing the sensitization on the potential of COVID-19/malaria co-infections. Considering that malaria tests are more readily available, it is advisable that health workers perform tests for malaria as they screen for COVID-19. This problem is particularly more relevant for travellers and people in malaria-endemic countries. It presents a chance to respond to two infectious diseases timely and reduce avoidable complications and deaths.

## How COVID-19 can affect the implementation of the malaria programmes

Malaria is a widely endemic disease in sub-Saharan Africa. Malaria control measures, which are delivered through public health facilities, prevent nearly 100 million new cases per year and save about 600,000 lives. The outbreak of COVID-19 can lead to disturbance in health delivery systems, inappropriate treatment and untreated malaria cases, resulting in an increase in mortality and morbidity. Due to the health delivery system disruption during Ebola virus outbreak, the number of deaths from malaria exceeded the number of deaths due to Ebola virus [66]. Assuring access to core malaria prevention measures is an essential approach to reduce strain on health systems; these involve vector control measures, such as insecticide-treated nets (ITNs) and indoor residual spraying, in addition to chemoprevention for pregnant women and young children (intermittent preventive treatment in pregnancy (IPTp), intermittent preventive treatment in infants (IPTi) and seasonal malaria chemoprevention(SMC)). Extra measures that could also reduce the burden on health systems in the setting of COVID-19 include presumptive malaria treatment and mass drug administration. (Specific considerations of community-based malaria care, including outreach and campaigns, in the context of the COVID-19 pandemic are outlined in Table 1.)

**Table 1:** Specific considerations of community-based malaria care, including outreach and campaigns, in the context of the COVID-19 pandemic

Access to and use of one of the core vector control tools should be maintained (ITNs or indoor residual spraying), including through adapted campaigns that are delivered using best practices to protect health workers and communities from COVID-19. Modifications might include canceling some data and accountability procedures that increase person-to-person contact and the potential risk for COVID-19 transmission (for example, not requiring a signature for ITNs received by a household)
Campaigns for seasonal malaria chemoprevention should proceed
Countries where malaria has been eliminated and those working to prevent re-establishment should maintain intensive malaria community-based surveillance activities in addition to core vector control activities, using best practices to safeguard health workers and communities
In exceptional situations, such as when there is a significant deterioration or inability of the health system to deliver services, mass administration of anti-malarial treatment could be used to rapidly reduce mortality and morbidity
Countries should consider increasing efforts to inspect and treat malaria, including at the community level, such as through community integrated management of childhood illness, especially during the country-based malaria seasons
Countries should continuously monitor and re-evaluate at regular intervals the necessity for delaying community-based surveys, mass treatment and active case finding based on the data provided
Community-based vector control and public health interventions should continue with strict precautions (hand hygiene, respiratory etiquette, physical distancing) observed by all participants in areas where there is no community transmission of COVID-19
In areas with community transmission, only essential activities should be continued. For vector control, essential activities should be interpreted as source reduction of vector breeding sites in and around houses
In areas that are affected by malaria and under stay-at-home measures due to COVID-19, families could work together for 30 min every week to get rid of potential mosquito breeding sites, clean roof gutters and ensure that all water storage containers are covered
Community-based WASH activities should continue, with amendments to include key information about preventing COVID-19 in settings where there are no cases of COVID-19. In settings where COVID-19 transmission is occurring, WASH messages should be repurposed to focus on preventing COVID-19 transmission

There are two main modalities to deliver these interventions. The insecticide-treated bed nets and seasonal malaria chemoprevention are delivered through population-wide campaigns while other interventions are delivered through patient/client-care mode. The implementation of these malaria programs is affected by travel restrictions, curfew, and the lockdown imposed during the COVID-19 pandemic. The enactment of these programs must consider the importance of both reducing malaria-related deaths and maintaining the safety of communities and health care providers. Activities should be organized in a way that avoids the gathering of people without abiding by the precautions for personal protection. Activities that increase the risk of COVID-19 or are difficult to implement without breaking protective measures should be stopped [67]. To ensure the continuity of country-based malaria program services, the national Malaria programs should adopt the COVID19-related recommendations that enhance the delivery of malaria control services with ensuring the safety of clients, patients, MoH personnel and service delivery teams, while continuing malaria prevention and case management activities to the greatest extent possible, i.e.: continuing to implement core vector control activities to the greatest extent possible, maintaining the continuity of access to care and active care-seeking for febrile illness and suspected malaria among the population, ensuring the appropriate testing and treatment of patients, and ensuring the delivery of existing programs involving the preventive use of antimalarial drugs among target populations by maintaining the agility of supply chain management, with a focus on pregnant women (delivering IPTp), children under 5 years of age in areas of highly seasonal malaria transmission (delivering SMC), and infants (delivering IPTi). Malaria prevention and treatment is even more important during the COVID-19 pandemic than under normal circumstances. In the face of that, all those procedures should be done while maintaining the safety of health workers and clients/patients in the context of COVID-19 transmission [68]. Finally, the national programs of malaria should also be ready to correct any misinformation, rumors, or misunderstanding like increasing the risk of contracting COVID-19 after using Chinese produced ITNs.

## Conclusion

In conclusion, COVID-19 has a variable prevalence among countries which is lower than expected in malaria-endemic regions. In addition to the possible role of health infrastructure and mitigation tools adopted, the variable distribution of the ACEI/D and the ACE2 (C1173T substitution) polymorphisms could partly explain this variable prevalence. Also, malaria patients develop anti-GPI antibodies which could identify SARS-CoV-2 glycoproteins and consequently play a protective role against COVID-191 or inducing a milder disease pattern. Both hydroxychloroquine (HCQ) and chloroquine (CQ) may have preventive and curative effects against SARS-CoV-2 virus through different mechanisms, however, clinical trials are still investigating the use of these medications as a potential treatment and preventive measures. The lower than expected number of cases detected in Africa suggests that the young age structure may be protective of severe and thus detectable cases. Considering the similarity of symptoms of malaria and COVID-19, clinicians may misdiagnose a malaria case as COVID-19 or vice versa or may overlook the possible coinfection. Finally, the lockdown and restricting movements of health care providers due to the COVID-19 pandemic has disturbed the continuation of malaria control programs such as the distribution of seasonal malaria chemoprevention and insecticide-treated bed nets resulting in more malaria cases and deaths.

## Data Availability

The data used in this report is available to readers.

## Abbreviations

HCQ: Hydroxychloroquine
CQ: Chloroquine
ITNs: Insecticide-treated nets
SARS-CoV-2: Severe acute respiratory syndrome coronavirus 2
WHO: World health organization
AFTCOR: African Task Force for Coronavirus Preparedness and Response
AU: The African Union
ANG II: Angiotensin II
ACE1: Angiotensin-converting enzyme 1
ACE2: Angiotensin-converting enzyme 1
ARDS: Acute respiratory distress syndrome
MERS: Middle East respiratory syndrome
GPI: Glycosylphosphatidylinositol
GPs: Glycoproteins
EBV: Epstein–Barr virus
HIV: Human immunodeficiency virus
TNF: Tumour necrosis factor
IL: Interleukins
DIC: Disseminated intravascular coagulation
SMC: Seasonal malaria chemoprevention
IPTp: Intermittent preventive treatment in pregnancy
IPT: Intermittent preventive treatment in infants

## References

[1] WHO. World malaria report 2019. Geneva: World Health Organization; 2019. https://www.who.int/publications/i/item/world-malaria-report-2019

[2] WHO declares public health emergency for novel coronavirus. https://www.medscape.com/viewarticle/924596

[3] WHO declares global emergency as Wuhan coronavirus spreads—The New York Times. https://www.nytimes.com/2020/01/30/health/coronavirus-world-health-organization.html

[4] WHO. Coronavirus disease (COVID-19). 2020. https://www.who.int/docs/default-source/coronaviruse/situation-reports/20200608-covid-19-sitrep-140.pdf?sfvrsn=2f310900_2

[5] Ondoa P, Kebede Y, Loembe MM, Bhiman JN, Tessema SK, Sow A (2020). COVID-19 testing in Africa: lessons learnt. Lancet Microbe.

[6] World Population Prospects—population division—United Nations. 2020. https://population.un.org/wpp/default.aspx?aspxerrorpath=/wpp/DataQuery/. Accessed 13 March 2020.

[7] Dowd JB, Andriano L, Brazel DM, Rotondi V, Block P, Ding X (2020). Demographic science aids in understanding the spread and fatality rates of COVID-19. Proc Natl Acad Sci USA.

[8] Mougeni F, Mangaboula A, Lell B (2020). The potential effect of the African population age structure on COVID-19 mortality. medRxiv.

[9] Zhou P, Yang XL, Wang XG, Hu B, Zhang L, Zhang W (2020). A pneumonia outbreak associated with a new coronavirus of probable bat origin. Nature.

[10] Rice GI, Thomas DA, Grant PJ, Turner AJ, Hooper NM (2004). Evaluation of angiotensin-converting enzyme (ACE), its homologue ACE2 and neprilysin in angiotensin peptide metabolism. Biochem J.

[11] Zhang H, Baker A (2017). Recombinant human ACE2: acing out angiotensin II in ARDS therapy. Crit Care.

[12] Silva LS, Silva-Filho JL, Caruso-Neves C, Pinheiro AAS (2016). New concepts in malaria pathogenesis: the role of the renin-angiotensin system. Front Cell Infect Microbiol.

[13] Hatami N, Ahi S, Sadeghinikoo A, Foroughian M, Javdani F, Kalani N (2020). Worldwide ACE (I/D) polymorphism may affect COVID-19 recovery rate: an ecological meta-regression. Endocrine.

[14] Delanghe JR, Speeckaert MM, De Buyzere ML (2020). The host’s angiotensin-converting enzyme polymorphism may explain epidemiological findings in COVID-19 infections. Clin Chim Acta.

[15] Saab YB, Gard PR, Overall ADJ (2007). The geographic distribution of the ACE II genotype: a novel finding. Genet Res.

[16] King T, Lamb T (2015). Interferon-γ: the Jekyll and Hyde of malaria. PLoS Pathog.

[17] Strayer D, Dickey R, Carter W (2014). Sensitivity of SARS/MERS CoV to interferons and other drugs based on achievable serum concentrations in humans. Infect Disord Drug Targets.

[18] Fauci AS, Lane HC, Redfield RR (2020). Covid-19—navigating the uncharted. N Engl J Med.

[19] de Mendonça VR, Barral-Netto M (2015). Immunoregulation in human malaria: the challenge of understanding asymptomatic infection. Mem Inst Oswaldo Cruz.

[20] Gomes LR, Martins YC, Ferreira-Da-Cruz MF, Daniel-Ribeiro CT (2014). Autoimmunity, phospholipid-reacting antibodies and malaria immunity. Lupus.

[21] Napoli PE, Nioi M (2020). Global spread of coronavirus disease 2019 and malaria: an epidemiological paradox in the early stage of a pandemic. J Clin Med.

[22] Savarino A, Boelaert JR, Cassone A, Majori G, Cauda R (2003). Effects of chloroquine on viral infections: an old drug against today’s diseases?. Lancet Infect Dis.

[23] Vincent MJ, Bergeron E, Benjannet S, Erickson BR, Rollin PE, Ksiazek TG (2005). Chloroquine is a potent inhibitor of SARS coronavirus infection and spread. Virol J.

[24] Dyall J, Gross R, Kindrachuk J, Johnson RF, Olinger GG, Hensley LE (2017). Middle east respiratory syndrome and severe acute respiratory syndrome: current therapeutic options and potential targets for novel therapies. Drugs.

[25] Yao X, Ye F, Zhang M, Cui C, Huang B, Niu P (2020). In vitro antiviral activity and projection of optimized dosing design of hydroxychloroquine for the treatment of severe Acute Respiratory Syndrome Coronavirus 2 (SARS-CoV-2). Clin Infect Dis.

[26] Mehra MR, Desai SS, Ruschitzka F, Patel AN (2020). Hydroxychloroquine or chloroquine with or without a macrolide for treatment of COVID-19: a multinational registry analysis. Lancet.

[27] The Lancet Editors (2020). Expression of concern: hydroxychloroquine or chloroquine with or without a macrolide for treatment of COVID-19: a multinational registry analysis. Lancet.

[28] Mehra MR, Ruschitzka F, Patel AN (2020). Retraction—hydroxychloroquine or chloroquine with or without a macrolide for treatment of COVID-19: a multinational registry analysis. Lancet.

[29] Ocan M, Akena D, Nsobya S, Kamya MR, Senono R, Kinengyere AA (2019). Persistence of chloroquine resistance alleles in malaria endemic countries: a systematic review of burden and risk factors. Malar J.

[30] Zhou D, Dai S-M, Tong Q (2020). COVID-19: a recommendation to examine the effect of hydroxychloroquine in preventing infection and progression. J Antimicrob Chemother.

[31] Amaravadi R. The PATCH Trial (Prevention and treatment of COVID-19 with hydroxychloroquine). ClinicalTrials.gov. 2020. https://www.clinicaltrials.gov/ct2/show/NCT04329923

[32] Lother SA, Abassi M, Agostinis A, Bangdiwala AS, Cheng MP, Drobot G (2020). Post-exposure prophylaxis or pre-emptive therapy for severe acute respiratory syndrome coronavirus 2 (SARS-CoV-2): study protocol for a pragmatic randomized-controlled trial. Can J Anesth.

[33] Boulware DR, Pullen MF, Bangdiwala AS, Pastick KA, Lofgren SM, Okafor EC (2020). A randomized trial of hydroxychloroquine as postexposure prophylaxis for Covid-19. N Engl J Med.

[34] NIAID. Evaluating the efficacy of hydroxychloroquine and azithromycin to prevent hospitalization or death in persons with COVID-19. ClinicalTrials.gov. https://clinicaltrials.gov/ct2/show/NCT04358068

[35] No clinical benefit from use of hydroxychloroquine in hospitalised patients with COVID-19. 2020. https://www.recoverytrial.net/files/hcq-recovery-statement-050620-final-002.pdf

[36] Lotteau V, Teyton L, Peleraux A, Nilsson T, Karlsson L, Schmid SL (1990). Intracellular transport of class II MHC molecules directed by invariant chain. Nature.

[37] Jang C-H, Choi J-H, Jue D-M (2006). Chloroquine inhibits production of TNF-a, IL-1b and IL-6 from lipopolysaccharide-stimulated human monocytes/macrophages by different modes. Rheumatology.

[38] Kužnik A, Benčina M, Švajger U, Jeras M, Rozman B, Jerala R (2011). Mechanism of endosomal TLR inhibition by antimalarial drugs and imidazoquinolines. J Immunol.

[39] Ewald SE, Lee BL, Lau L, Wickliffe KE, Shi GP, Chapman HA (2008). The ectodomain of Toll-like receptor 9 is cleaved to generate a functional receptor. Nature.

[40] Millet JK, Whittaker GR (2015). Host cell proteases: critical determinants of coronavirus tropism and pathogenesis. Virus Res.

[41] Schrezenmeier E, Dörner T (2020). Mechanisms of action of hydroxychloroquine and chloroquine: implications for rheumatology. Nat Rev Rheumatol.

[42] Al-Bari MAA (2017). Targeting endosomal acidification by chloroquine analogs as a promising strategy for the treatment of emerging viral diseases. Pharmacol Res Perspect.

[43] Guan W, Ni Z, Hu Y, Liang W, Ou C, He J (2020). Clinical characteristics of coronavirus disease 2019 in China. N Engl J Med.

[44] O’Brien D, Tobin S, Brown GV, Torresi J (2001). Fever in returned travelers: review of hospital admissions for a 3-year period. Clin Infect Dis.

[45] Gostic KM, Gomez ACR, Mummah RO, Kucharski AJ, Lloyd-Smith JO (2020). Estimated effectiveness of symptom and risk screening to prevent the spread of COVID-19. Elife.

[46] Huang C, Wang Y, Li X, Ren L, Zhao J, Hu Y (2020). Clinical features of patients infected with 2019 novel coronavirus in Wuhan. China Lancet.

[47] Ye Q, Wang B, Mao J (2020). The pathogenesis and treatment of the ‘Cytokine Storm’ in COVID-19. J Infect.

[48] Liao Y-C, Liang W-G, Chen F-W, Hsu J-H, Yang J-J, Chang M-S (2002). IL-19 induces production of IL-6 and TNF-α and results in cell apoptosis through TNF-α. J Immunol.

[49] Wu Z, McGoogan JM (2020). Characteristics of and important lessons from the Coronavirus Disease 2019 (COVID-19) outbreak in China: summary of a report of 72314 cases from the Chinese Center for Disease Control and Prevention. JAMA.

[50] Bucşan AN, Williamson KC (2020). Setting the stage: the initial immune response to blood-stage parasites. Virulence.

[51] Othoro C, Lal AA, Nahlen B, Koech D, Orago ASS, Udhayakumar V (1999). A low interleukin-10 tumor necrosis factor-α ratio is associated with malaria anemia in children residing in a holoendemic malaria region in Western Kenya. J Infect Dis.

[52] Akanmori BD, Kurtzhals JA, Goka BQ, Adabayeri V, Ofori MF, Nkrumah FK (2000). Distinct patterns of cytokine regulation in discrete clinical forms of *Plasmodium falciparum* malaria. Eur Cytokine Netw.

[53] Jin Y, Yang H, Ji W, Wu W, Chen S, Zhang W (2020). Virology, epidemiology, pathogenesis, and control of COVID-19. Viruses.

[54] Mazhar F, Haider N (2016). Respiratory manifestation of malaria: an update. Int J Med Res Health Sci.

[55] Visseren FL, Bouwman JJ, Bouter KP, Diepersloot RJ, de Groot PH, Erkelens DW (2000). Procoagulant activity of endothelial cells after infection with respiratory viruses. Thromb Haemost.

[56] Giannis D, Ziogas IA, Gianni P (2020). Coagulation disorders in coronavirus infected patients: COVID-19, SARS-CoV-1, MERS-CoV and lessons from the past. J Clin Virol.

[57] Klok FA, Kruip MJHA, van der Meer NJM, Arbous MS, Gommers DAMPJ, Kant KM (2020). Incidence of thrombotic complications in critically ill ICU patients with COVID-19. Thromb Res.

[58] Oxley TJ, Mocco J, Majidi S, Kellner CP, Shoirah H, Singh IP (2020). Large-vessel stroke as a presenting feature of Covid-19 in the young. N Engl J Med.

[59] Tang N, Li D, Wang X, Sun Z (2020). Abnormal coagulation parameters are associated with poor prognosis in patients with novel coronavirus pneumonia. J Thromb Haemost.

[60] Fox SE, Akmatbekov A, Harbert JL, Li G, Quincy Brown J, Vander Heide RS (2020). Pulmonary and cardiac pathology in African American patients with COVID-19: an autopsy series from New Orleans. Lancet Respir Med.

[61] Lippi G, Plebani M, Henry BM (2020). Thrombocytopenia is associated with severe coronavirus disease 2019 (COVID-19) infections: a meta-analysis. Clin Chim Acta.

[62] Angchaisuksiri P (2014). Coagulopathy in malaria. Thromb Res.

[63] Krishnan A, Karnad DR, Limaye U, Siddharth W (2004). Cerebral venous and dural sinus thrombosis in severe falciparum malaria. J Infect.

[64] Schwartz J, Musoke C, Ssendikadiwa C, Babua C (2014). Severe falciparum malaria associated with massive pulmonary embolism. Ann Afr Med.

[65] Srichaikul T (1993). Hemostatic alterations in malaria. Southeast Asian J Trop Med Public Health.

[66] Plucinski M, Butts JK, Halsey ES, Mcelroy PD, Aboulhab J, Plucinski MM (2015). Effect of the Ebola-virus-disease epidemic on malaria case management in Guinea, 2014: a cross-sectional survey of health facilities. Lancet Infect Dis.

[67] WHO. Responding to community spread of COVID-19. Interim Guide 7 March. 2020;1–6. https://www.who.int/publications/i/item/responding-to-community-spread-of-covid-19

[68] Tailoring malaria interventions in the COVID-19 response. https://www.who.int/publications/m/item/tailoring-malaria-interventions-in-the-covid-19-response

